# Angiotensin II Inhibits Adipogenic Differentiation and Promotes Mature Adipocyte Browning through the Corepressor CtBP1

**DOI:** 10.3390/biomedicines10123131

**Published:** 2022-12-04

**Authors:** Xiuying Liang, Jingwen Sun, Haijing Guan, Qingyu Zhu, Wenjuan Yao

**Affiliations:** School of Pharmacy, Nantong University, 19 QiXiu Road, Nantong 226001, China

**Keywords:** adipogenesis, CtBP1, angiotensin II, mitochondria, ROS

## Abstract

The mechanisms of angiotensin II (Ang II) on regulating adipogenic differentiation and function remain unknown. In this study, we focus on revealing the role of C-terminal-binding protein 1 (CtBP1) on Ang II-mediated adipogenic differentiation and mature adipocyte browning. Amounts of 3T3-L1 and CtBP1-KO 3T3-L1 were treated with Ang II for 24 h and then induced adipogenic differentiation, or cells were first induced differentiation and then treated with Ang II. The expressions of CtBP1 and adipogenic markers were checked by Western blot. Transcription of CtBP1 was assayed by Real-time RT-PCR. Lipid droplet formation and size were detected by Oil Red O. Mitochondrial content and reactive oxygenspecies (ROS) were detected by Mito-tracker and MitoSOX. Mitochondrial respiratory function was detected with the corresponding kits. Mitochondrial membrane potential (MMP) (∆Ψm) was assayed by JC-1. The results show that Ang II promoted CtBP1 transcription and expression via AT1 receptor during 3T3-L1 adipogenic differentiation. Ang II significantly inhibited lipid droplet formation and adipogenic markers expression in 3T3-L1 differentiation, which was blocked by CtBP1 knockout. In mature 3T3-L1, Ang II treatment increased uncoupling protein-1 (UCP-1) expression and the number of lipid droplets, and also reduced lipid droplet size and single cell lipid accumulation, which was reversed by CtBP1 knockout. In addition, Ang II treatment enhanced mitochondrial numbers, ATP production, oxygen consumption rate (OCR) and ROS generation, and reduced MMP (∆Ψm) via CtBP1 in mature 3T3-L1 adipocytes. In conclusion, this study demonstrates that CtBP1 plays a key role in the inhibitory effect of Ang II on adipogenesis. Moreover, Ang II regulates the function of mature adipocyte via CtBP1, including promoting adipocyte browning, mitochondrial respiration and ROS generation.

## 1. Introduction

Adipose tissue plays a principal role in overall metabolic homeostasis, and its dysfunction can lead to metabolic disorders, most notably type 2 diabetes and cardiovascular disease [1]. Mature adipocytes express all components of the renin–angiotensin system (RAS), including angiotensin II (Ang II), and also express the type 1 (AT1) and type 2 (AT 2) angiotensin receptor subtypes [2]. For cardiovascular disease, perivascular adipose tissue (PVAT) is a specific adipose tissue and now recognized to be an endocrine organ with important physiological and pathological effects on vascular homeostasis and vascular diseases [3]. There are many studies showing that PVAT dysfunction may induce several pathophysiological states such as obesity, atherosclerosis, or hypertension [3,4,5]. We previously reported that Ang II significantly changed thoracic PVAT (tPVAT) phenotype, making it irregular, and hypertrophy, which suggests that excessive activation of local RAS can lead to adipose tissue dysfunction [6]. Since adipocytes are the main cellular component of adipose tissue, we speculated that Ang II may affect the differentiation and function of adipocytes. It has been reported that Ang II inhibits the differentiation of preadipocytes via AT1 receptor in mice and humans [7,8]. However, the exact mechanisms by which Ang II regulates adipogenic differentiation remain unclear.

There are essentially two major types of adipose tissue with different functions: brown adipose tissue (BAT) containing multiple lipid droplets and densely packed mitochondria, and white adipose tissue (WAT) with large lipid droplets and relatively low expression levels of UCP-1 (mitochondrial uncoupling protein-1) [3]. The function of WAT is mainly to store fat, while that of BAT is mainly to burn fat for heat production. WAT and BAT regulate overall energy balance by affecting the function of other tissues [9]. It has been reported that knockdown of ribosomal protein S3A (RPS3A) significantly impairs the function of mitochondria in mature adipocytes [10]. Elective-activation of estrogen receptor alpha (ERα) in adipocytes induces beiging of adipocytes and promotes brown adipocyte characteristics [11]. In addition, Fst (follistatin) promotes brown adipocyte characteristics in both WAT and BAT depots in vivo through distinct mechanisms [12]. However, there is currently no report showing the effects and related mechanisms of Ang II on regulating the function of mature adipocytes.

C-terminal-binding protein (CtBP) mainly acts in the nucleus as a transcriptional co-repressor and a metabolic sensor that is activated by NADH [13]. CtBP proteins (CtBP1 or CtBP2) are expressed at high levels during development, participating in axial-patterning, cellular proliferation, and differentiation [14]. It has been reported that PR domain-containing protein 16 (PRDM16) and its associated coregulators peroxisome proliferator activated receptor γ (PPARγ), coactivator-1α (PGC-1α) and CtBP1/2 control brown adipocyte formation and function [15]. In addition, C/EBPα and the corepressors CtBP1 or CtBP2 inhibit the expression of white adipocyte genes [16]. Therefore, we speculated that CtBP may be involved in regulating the function of mature adipocytes. In this study, we focus on revealing the effects of CtBP1 on adipogenic differentiation, brown adipocyte formation, and mitochondrial function in Ang II-induced 3T3-L1 adipocytes.

## 2. Materials and Methods

### 2.1. Materials

Angiotensin II (Ang II; HY-B0202) and Candesartan (AT1 receptor inhibitor; CC1734) were purchased from MedChemExpress (Princeton, NJ, USA) and ChemCatch Co., Ltd. (Shanghai, China), respectively. Insulin (human) recombinant expressed in yeast (B7407), rosiglitazone (A4304) and dexamethasone (DHAP) (A2324) were bought from APEx-BIO (Shanghai, China). The primary antibodies against GAPDH (#5174) and β-tubulin (AT0003) were purchased from Cell Signaling Technology (Beverly, MA, USA) and CMCTAG Inc (San Diego, CA, USA), respectively. Polyclonal antibodies against peroxisome proliferator-activated receptor (PPAR) γ (AF7797) and adipocyte fatty-acid-binding protein (AP2 or FABP4) (AF6843), and monoclonal antibody against acetyl CoA carboxylase (ACC) (AF1867) were purchased from Beyotime Biotechnology (Shanghai, China). Monoclonal antibodies against C-terminal-binding protein 1 (CtBP1) (ab129181), CCAAT enhancer-binding protein alpha (C/EBPα) (ab40764) were purchased from Abcam (Cambridge, UK). Peroxidase conjugated affinipure goat anti-rabbit IgG (H + L) (SA00001-2) and HRP-conjugated goat anti-mouse IgG (D110087-0100) were purchased from Proteintech (Chicago, IL, USA) and BBI Life Sciences (Hongkong, China), respectively. JC-1 staining buffer (C2003S-2) and JC-1 staining solution (C2003S-1) were bought from Beyotime Biotechnology (Shanghai, China). RNA simple Total RNA Kit and BeyoFastTM SYBR Green Qpcr Mix (2X, High ROX) were purchased from TIANGEN Biotech (Beijing, China) and Beyotime Biotechnology (Shanghai, China), respectively. The BCA Protein Assay Kit (CW0014S) was purchased from CW Biotech (Beijing, China). DMEM medium and fetal bovine serum (FBS; F2442) were obtained from Sigma Chemical Co., (St. Louis, MO, USA). DMEM complete medium (ZQ-107) containing 10% calf serum and 1% penicillin-streptomycin (P/S) was purchased from Zhong Qiao Xin Zhou Biotechnology Co., Ltd. (Shanghai, China). DAPI staining solution (C1005), ATP assay kit (S0026), Mito-Tracker Green (C1048), and oil red O staining solution (E607319) were purchased from Beyotime Biotechnology (Shanghai, China). MitoSOX red mitochondrial superoxide indicator (40778ES50) was purchased from YEASEN Biotech Co., Ltd. (Shanghai, China). Oxygen Consumption Assay Kit/Oxygen Consumption Rate Assay Kit/OCR Assay Kit was bought from BBoxiProbe^®^ (BB-48211, Shanghai, China). Other chemicals used in this study were of analytical grade and were made in China.

### 2.2. Cell Culture and Treatment

The 3T3-L1 and CtBP1-KO 3T3-L1 cells were obtained from Cyagen Biosciences Inc (Guangdong, China). They were cultured in DMEM containing 10% newborn calf serum and 1% P/S solution at 37 °C in a humidified atmosphere containing 5% CO_2_. The process of differentiation of 3T3-L1 into mature adipocytes is as follows: after seeding, the confluent cells were added to DMEM with 10% FBS, 10 μg/mL insulin, 0.25 mM dexamethasone, 0.5 mM IBMX (3-isobutyl-1-methylxanthine), and 1 μM rosiglitazone. After two days, the medium was changed to DMEM with 10% FBS and 10 μg/mL insulin for the next 48 h. Then the cells were cultured with maintenance medium (DMEM containing 10% FBS) for the next 96 h. The degree of cell differentiation was detected by Oil Red O staining.

In order to observe the effects of Ang II and its receptor on differentiation of 3T3-L1 preadipocyte into mature adipocyte, the confluent cells were pretreated with 5 μM candesartan (AT1 receptor inhibitor) for 6 h and then 100 nM Ang II for 24 h, and finally the differentiation medium was added to the cells to repeat the above differentiation process (Figure S1). In order to observe the effects of Ang II and its receptor on the function of mature adipocytes, the cells were treated with candesartan and Ang II after the differentiation process (Figure S2).

### 2.3. Construction of CtBP1-KO 3T3-L1 Cell Line

The mouse CtBP1 gene was knocked out by CRISPR/Cas9 gene editing technology completed in Cyagen Company. After electroporation, the single cell clone was selected and verified by PCR and sequencing. The gRNA sequences were as follows: gRNA-A1: GGGACGACAGGCGCCACAGT-TGG; gRNA-A2: GCTAGATTTGACCTGTCTAG-GGG; gRNA-B1: CTGCCACTCCCAATCTAATG-TGG; gRNA-B2: CACAGGGGCCACAACCTTGG-AGG. The primer sequences used to verify the CtBP1 KO site were as follows: Region 1, upstream: TGTAGCTGTAGAAAGGGAGGCT, downstream: GAGAGAAAACAGGCTGCTCACTA; Region 2, upstream: TAACTTTACTCACTGCGTTGTCAC, downstream: TGACACAAGGAGGAACCAAGAAAG; Region 3, upstream: TGTAGCTGTAGAAAGGGAGGCT, downstream: TGACACAAGGAGGAACCAAGAAAG. Finally, we verified the knockout of CtBP1 by Western blotting.

### 2.4. Oil Red O Staining

After treatment, cellular lipid droplet formation and size were detected by Oil Red O staining. After removing the medium, cells were gently washed with 400 μL PBS. Then cells were added with 200 μL 10% neutral buffered formalin and fixed for 30 min. After that, cells were washed with 400 μL PBS and added into 200 μL 60% isopropanol (100% isopropanol: ddH_2_O = 3:2) for 10 s. Finally, cells were added into 200 μL Oil Red O working solution and incubated at room temperature for 1 h. Oil Red O working solution was filtered with 0.45 μm filter membrane and left at room temperature for 10 min in order to remove the impurities and make the dyeing result clearer. After removing the staining solution, cells were washed with distilled water and observed under an inverted microscope. Lipid accumulation was measured in three fields per sample using ImageJ software v1.8.0 (Labworks, Maryland, MD, USA).

### 2.5. Western Blot Analysis

Cells were lysed for 40 min in RIPA lysis buffer containing protease and phosphatase inhibitor. The supernatant was collected and put on ice for 40 min, and then centrifuged at 4 °C for 15 min. Protein concentrations were measured using BCA Protein Assay Kit. The lysate was diluted with 5 × SDS-polyacrylamide gelelectrophoresis (PAGE) loading buffer and heated at 100 °C for 10 min. The protein samples were separated by 6–12% SDS-PAGE and electrotransferred onto nitrocellulose membranes. The membranes were then blocked with 5% fat free milk for 2 h at room temperature and incubated with primary antibodies overnight at 4 °C after washing with PBS. Antibody–antigen binding was checked by incubation with HRP-conjugated secondary antibodies for 2 h at room temperature. The band signals were detected by ECL Protein Western Blot Detection Kit (Amersham Pharmacia, Little Chalfont, Bucks, UK) and Image Quantlas 500 (GE Healthcare Bio-Sciences AB, Uppsala, Sweden). GAPDH and β-tubulin were used as internal standards.

### 2.6. Real-Time RT-PCR

Total RNA was isolated using a Total RNA Purification Kit in accordance with the manufacturer’s instructions. One microgram of RNA was reverse transcribed using a first-strand cDNA synthesis kit. The cDNA was then mixed with Maxima SYBR Green qPCR Master Mix and used as a template for PCR with gene specific primers (CtBP1: F: 5′-GGAGATCCATGAGAAGGTACTG-3′, R: 5′-GATGTCGATATTGTCAAACCCG-3′; GAPDH: F: 5′-ACAACTCTCTCAAGATTGTCAGCAA-3′, R: 5′-ACTTTGTGAAGCTCATTTCCTGG-3′). The amplification conditions used for PCR cycling were as follows: 94 °C for 30 s, 58 °C for 35 s, 72 °C for 35 s, 35 cycles (CtBP1 and GAPDH). Quantitative PCR was performed using a Corbett RG-6000 Real-time PCR system (Corbett Life Sciences, Mortlake, NSW, Australia). Relative expression levels were determined after normalization to GAPDH. The 2^−ΔΔCt^ method was applied to calculate the relative expression level of CtBP1.

### 2.7. Detection of Mitochondrial ROS

Mito-tracker green was prepared with DMSO to a final concentration of 1 mM and was then diluted with HBSS buffer to a final concentration of 200 nM. The mitochondrial content in mature 3T3-L1 cells was detected by Mito-tracker green. Relative fluorescence intensity of mitochondria was measured in three fields for each sample using ImagePro Plus software 6.0 (Media Cybernetics, Maryland, MD, USA). For mitochondrial ROS detection, 5 mM of MitoSOX red mitochondrial superoxide indicator storage solution was diluted to a final concentration of 5 µM with HBSS. Mito-tracker green and MitoSOX red working solution were added to the cells and then incubated at 37 °C for 15 min in the dark. Cells were washed and added to DAPI at 37 °C for 15 min in the dark and then observed under a confocal laser scanning microscope (FV500; Olympus, Tokyo, Japan) or fluorescence microscope (Nikon, Tokyo, Japan).

### 2.8. Mitochondrial Content Assay

The mature adipocyte cells were treated with Ang II for 24 h. The mitochondrial content in adipocytes were determined by Mito-Tracker Green. Fluorescent images were observed and captured with a fluorescence microscope (Nikon, Tokyo, Japan). Relative fluorescence intensity of mitochondria was measured in three fields per sample using ImageJ software v1.8.0 (Labworks, Bethesda, MD, USA).

### 2.9. Detection of Mitochondrial Respiratory Function

For ATP production assay, ATP Assay kit was used according to the manufacturer’s instructions. Cells were lysed and centrifuged at 4 °C for 15 min at 12,000× *g*. The supernatant was added to 100 μL ATP detection working solution containing luciferase. The luminance (RLU) value of the mixture was measured with a microplate-based luminometer (Bio Tek, Winooski, VT, USA). The concentration of ATP was calculated according to a standard curve and normalized using the cellular protein level.

For oxygen consumption assay, oxygen consumption was assayed with Oxygen Consumption Assay Kit/Oxygen Consumption Rate Assay Kit/OCR Assay Kit according to the manufacturer’s instructions. Briefly, cells to be detected were mixed with 10 μL BBoxiProbeTM R01 oxygen fluorescence probe, and then 100 μL of oxygen sealing solution was added to prevent external oxygen generation. The fluorescence light intensity was then measured with a luminometer (excitation 468 nm; emission 603 nm) over 2 h at 2-min intervals. The rate of oxygen consumption was calculated as follows: oxygen consumption rate (OCR; %) = (final fluorescence–initial fluorescence)/(final fluorescence in controls−initial fluorescence in controls) × 100%.

### 2.10. Detection of Mitochondrial Membrane Potential (∆Ψm)

The treated cells were gently rinsed with JC-1 staining buffer once or twice, and then incubated with JC-1 staining solution for 20 min at 37 °C. Subsequently, cells were washed with JC-1 staining buffer for 2~3 times and incubated with DAPI for 15 min at 37 °C. Images were observed and captured under a fluorescence microscope (Nikon, Tokyo, Japan). The red fluorescence indicates JC-1aggregates, and the green fluorescence indicates JC-1 monomers.

### 2.11. Statistical Analysis

All the results are expressed as the mean ± SD. One-way ANOVA followed by Tukey’s post-hoc test, as implemented in SPSS V22.0 (IBM, New York, NY, USA), were used for statistical analysis. Differences with a value of *p* < 0.05 were considered to be statistically significant.

## 3. Results

### 3.1. Ang II Inhibits 3T3-L1 Adipogenicity via CtBP1

**Figure 1:**
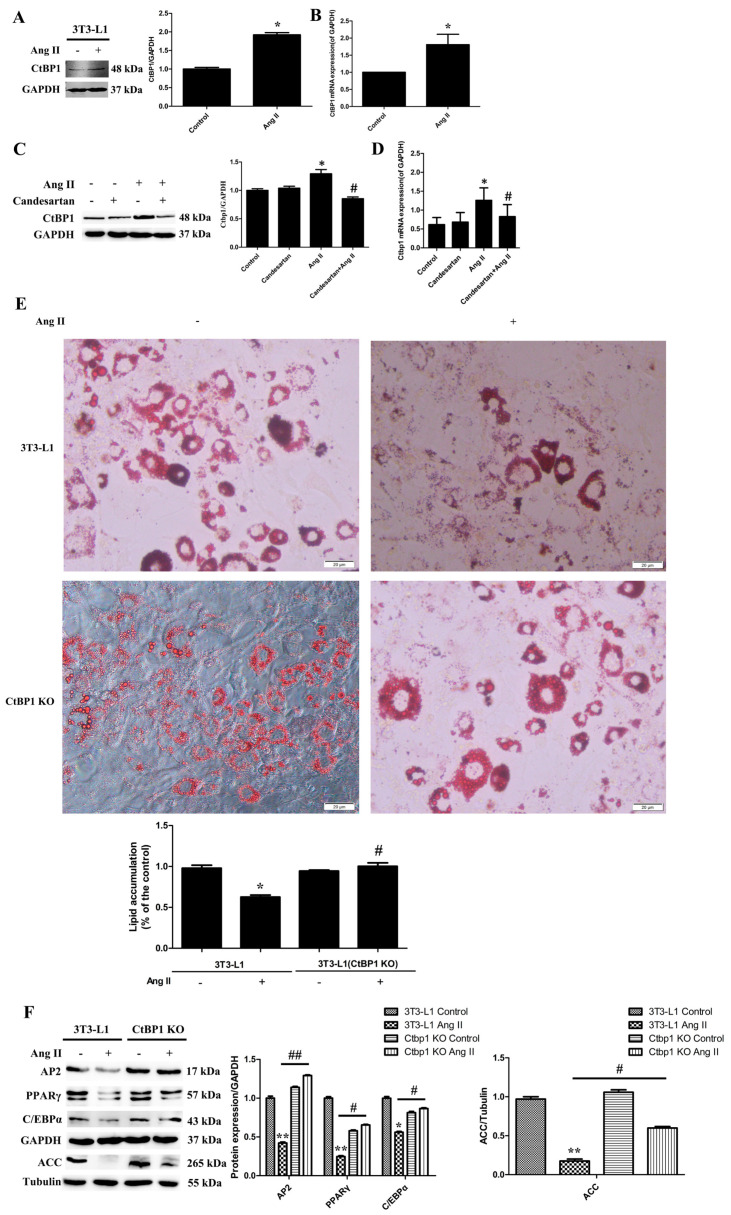
Ang II promotes the expression of CtBP1 through the AT1 receptor, and thereby inhibiting the process of 3T3−L1 adipogenic differentiation. The 3T3−L1 wild-type or CtBP1 KO cells were pretreated with 5 μM candesartan (AT1 receptor inhibitor) for 6 h and then 100 nM Ang II for 24 h, and finally underwent the adipogenic differentiation process. Untreated 3T3−L1 wild-type cells were used as control. (**A**) The effect of Ang II on CtBP1 expression during adipogenic differentiation detected by Western blot analysis. Histogram shows the ratio of CtBP1 to GAPDH. * *p* < 0.05 vs. the control group (*n* = 3). (**B**) Real-time RT−PCR analysis showed that Ang II significantly increased CtBP1 mRNA level. Histogram shows the ratio of CtBP1 mRNA level to GAPDH mRNA level. * *p* < 0.05 vs. the control group (*n* = 3). (**C**) The effect of candesartan treatment on Ang II−induced CtBP1 expression using Western blot analysis. Histogram shows the ratio of CtBP1 to GAPDH. * *p* < 0.05 vs. the control group; #, *p* < 0.05 vs. the Ang II−treated group (*n* = 3). (**D**) The effect of candesartan on Ang II−induced CtBP1 transcription using Real-time RT−PCR analysis. Histogram shows the ratio of CtBP1 mRNA level to GAPDH mRNA level. * *p* < 0.05 vs. the control group; #, *p* < 0.05 vs. the Ang II−treated group (*n* = 3). (**E**) The degree of cell adipogenicity was detected by Oil Red O staining. Ang II treatment significantly reduced the number and content of red lipid droplets and inhibited 3T3-L1 adipogenesis, which was blocked by CtBP1 knockout. Histogram shows lipid accumulation in cells. * *p* < 0.05 vs. the control group; #, *p* < 0.05 vs. the Ang II−treated wild type cells (*n* = 3). (**F**) The effect of Ang II on the expression of adipogenic markers (AP2, PPARγ, C/EBPα, ACC) in 3T3−L1 wild type and CtBP1 KO cells. Histograms show the ratios of target proteins to GAPDH or tubulin. * *p* < 0.05 and ** *p* < 0.01 vs. the control group; # *p* < 0.05 and ## *p* < 0.01 vs. Ang II−treated wild type cells (*n* = 3).

It has been reported that Ang II reduced adipose conversion by Ang II type 1 receptor, whereas blockade of this receptor markedly enhanced adipogenesis in both human and 3T3-L1 preadipocytes [7,8]. In this study, we also examined the effect of Ang II and its receptor on the expression of key adipogenic markers [17] such as PPARγ, AP2, C/EBPα, and ACC, during the adipogenic process of 3T3-L1 induced by Ang II. As shown in Figure S3, Ang II inhibited the expression of adipogenic markers in 3T3-L1 through an AT1 receptor, which is consistent with the previous reports. This also indicated that our 3T3-L1 adipogenic model was successfully constructed. However, the mechanism by which Ang II inhibited cell adipogenicity is still unclear, especially, the role of C-terminal binding protein 1 (CtBP1) on it has not been reported. To clarify the role of CtBP1 in the inhibition of 3T3-L1 adipogenic differentiation by Ang II, we analyzed the protein and mRNA levels of CtBP1 by Western blotting and real-time RT-PCR, respectively. As shown in Figure 1A,B, Ang II pretreatment significantly increased both the protein level and mRNA level of CtBP1 during 3T3-L1 adipogenesis. This indicates that Ang II may inhibit 3T3-L1 adipogenesis by promoting CtBP1 expression. In addition, Figure 1C,D shows that blockade of AT1 receptor significantly inhibited Ang II-induced CtBP1 transcription and expression. In other words, Ang II promotes CtBP1 expression through the AT1 receptor. To further confirm the effect of CtBP1 on 3T3-L1 adipogenesis, we constructed CtBP1-KO 3T3-L1 cell line by CRISPR/Cas9 gene editing technology. Figure S4 shows that CtBP1 was successfully knocked out in 3T3-L1 wild type cells. Oil red O staining shows that Ang II pretreatment significantly inhibited the formation of lipid droplets and lipid accumulation in 3T3-L1 wild-type cells, which could be reversed by CtBP1 knockout (Figure 1E). In CtBP1-KO 3T3-L1 cells, Ang II could not inhibit the formation of lipid droplets and mature adipocytes (Figure 1E). Furthermore, CtBP1 knockout dramatically increased the expression of adipogenic markers when compared with Ang II-treated 3T3-L1 wild-type cells (Figure 1F). In addition, CtBP1-KO cells without Ang II pretreatment successfully differentiated into mature adipocytes and expressed adipogenic proteins, which means that CtBP1 knockout by itself had no significant effect on 3T3-L1 adipogenicity.

### 3.2. Ang II Regulates the Browning and Mitochondrial Function of Mature 3T3-L1 through CtBP1

**Figure 2:**
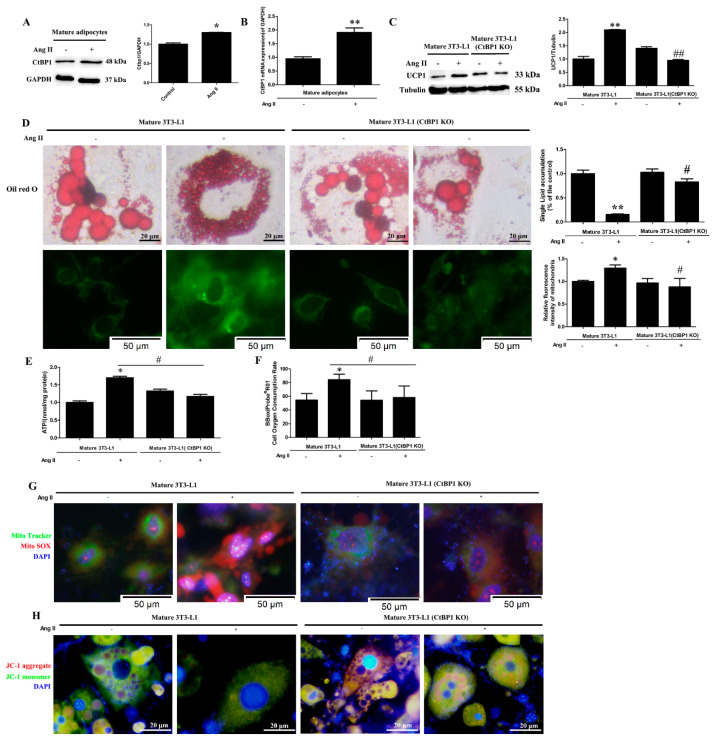
Ang II regulates adipocyte browning and mitochondrial function through CtBP1 in mature 3T3−L1 cells. The 3T3−L1 wild-type or CtBP1 KO cells firstly underwent the adipogenic differentiation process and were then treated with 100 nM Ang II for 24 h. Mature 3T3−L1 wild-type cells without Ang II treatment were used as control. (**A**) The effect of Ang II on CtBP1 expression in mature 3T3-L1 wild−type cells was detected by Western blot analysis. Histogram shows the ratio of CtBP1 to GAPDH. * *p* < 0.05 vs. the control group (*n* = 3). (**B**) The effect of Ang II on CtBP1 transcription in mature 3T3−L1 adipocytes using Real-time RT−PCR analysis. Histogram shows the ratio of CtBP1 mRNA level to GAPDH mRNA level. ** *p* < 0.01 vs. the control group (*n* = 3). (**C**) The effect of Ang II on the expression of UCP−1 in mature 3T3−L1 wild type and CtBP1 KO cells. Histogram shows the ratio of UCP−1 to tubulin. ** *p* < 0.01 vs. the control group; ## *p* < 0.01 vs. Ang II−treated mature 3T3-L1 wild type cells (*n* = 3). (**D**) The effect of Ang II on lipid droplet size and mitochondrial number detected by Oil Red O staining and Mito Tracker staining (green), respectively. Ang II treatment reduced lipid droplet size and single cell lipid accumulation and increased mitochondrial numbers which were blocked by CtBP1 knockout. Histograms show single cell lipid accumulation or relative fluorescence intensity of mitochondria. * *p* < 0.05 and ** *p* < 0.01 vs. the control group; # *p* < 0.05 vs. Ang II−treated mature 3T3−L1 wild type cells (*n* = 3). (**E**) The effect of Ang II on intracellular ATP concentration detected by an ATP Assay Kit. Histogram shows ATP levels in cells. * *p* < 0.05 vs. the control group; # *p* <0.05 vs. Ang II-treated mature 3T3−L1 cells (*n* = 3). (**F**) The effect of Ang II on oxygen consumption rat (OCR) detected by an Oxygen Consumption Rate Assay Kit. Histogram shows OCR (%) in each group. * *p* < 0.05 vs. the control group; # *p* < 0.05 vs. Ang II−treated mature 3T3−L1 cells (*n* = 3). (**G**) The effect of Ang II on mitochondrial ROS production. Mitochondrial superoxide was detected with MitoSOX red. Mitochondrial localization of the MitoSOX signal was confirmed by MitoTracker green. Nuclei were stained with DAPI (blue). (**H**) The effect of Ang II on MMP (∆Ψm) measured by JC−1 staining. The nuclei were stained with DAPI (blue).

Until now, the effect of Ang II on the function of mature adipocyte is unclear. Therefore, we treated cells with Ang II after 3T3-L1 differentiation into mature adipocytes in order to observe whether Ang II affects mature adipocyte function and illustrate the role of CtBP1 on it (Figure S2). Figure 2A,B shows that Ang II treatment promoted the protein and mRNA levels of CtBP1 in mature 3T3-L1 cells. In addition, we found that Ang II significantly increased the expression of UCP-1 (brown adipocyte marker) when compared with Ang II untreated cells (Figure 2C). These results indicate that Ang II may induce the browning of mature 3T3-L1 cells and CtBP1 may be involved in it. To further verify the effect of Ang II on the browning and function of mature 3T3-L1, we next observed lipid droplet morphology and mitochondrial indicators. Figure 2D shows that Ang II treatment significantly reduced the size of lipid droplets and single cell lipid accumulation, and increased the number of small lipid droplets, which is consistent with the characteristics of brown adipocytes and the results of increased UCP-1 expression. Since the primary function of brown adipocyte is to burn fat for heat production, it is necessary to measure mitochondrial function. Figure 2D shows that Ang II increased mitochondrial numbers (green fluorescence intensity) in mature 3T3-L1. Furthermore, Ang II dramatically enhanced the ATP production and oxygen consumption rate (OCR), indicating that Ang II can enhance the respiratory function of mature adipocytes (Figure 2E,F). Figure 2G,H shows that Ang II treatment increased the production of mitochondrial ROS (mROS) and decreased the MMP (∆Ψm) in mature 3T3-L1 cells, suggesting that oxidative stress and membrane potential levels of mature adipocytes can be regulated by Ang II. To further confirm whether CtBP1 is participate in the effects of Ang II on the browning and mitochondrial function of mature adipocytes, we found that CtBP1 knockout significantly reduced UCP-1 expression, lipid droplet and mitochondrial numbers, ATP production, OCR, mROS production, and increased lipid droplet size and MMP (∆Ψm) in mature 3T3-L1. These results establish that CtBP1 is involved in the effects of Ang II on the browning and mitochondrial function of mature adipocytes.

## 4. Discussion

C-terminal binding protein 1 (CtBP1) has been linked to multiple biological processes through its association with numerous transcription factors [18]. It has been reported to be involved in oncogenic processes and effective recycling of synaptic vesicles [19,20,21,22,23]. We have recently reported that CtBP1 is one of the top 10 up-regulated proteins during Ang II-induced PVAT pathogenesis [6]. In addition, some reports show that CtBP1/2 is involved in controlling the switch from WAT to BAT [15,16]. Therefore, we speculate that CtBP1 may be involved in the regulation of Ang II-induced adipocyte dysfunction. Since 3T3-L1 mouse preadipose cell line is a classic cell model for studying adipocyte differentiation and function, we used the 3T3-L1 model in this study to elucidate the role of CtBP1 in Ang II-treated adipocytes.

It has been reported that Ang II-AT1 signaling inhibits adipocyte differentiation and maturation [7,8]. In this study, our experiments also confirmed this conclusion (Figure 1 and Figure S3). However, the mechanism and molecular target of the inhibitory effect of Ang II on lipogenesis remain unclear. In this study, we found that Ang II promoted the transcription and expression of CtBP1 through AT1 receptor during Ang II-induced inhibition of 3T3-L1 differentiation (Figure 1A–D). We suggest that the increased CtBP1 is responsible for the inhibition effect of Ang II. By knockout of CtBP1 in 3T3-L1, we found that CtBP1 KO significantly reversed the inhibitory effect of Ang II on lipogenesis and promoted the formation of lipid droplets and the expression of adipogenic markers (Figure 1E,F). These results indicate that Ang II promotes the expression of CtBP1 through the AT1 receptor, and thereby inhibits the process of 3T3-L1 adipogenic differentiation. For the first time, we revealed that the inhibitory effect of Ang II-AT1 signaling on lipogenesis is dependent on CtBP1, which may be an important target for regulating adipocyte differentiation and maturation. In the future, we will continue to explore the specific mechanisms of CtBP1 in regulating the expression of adipogenic markers.

Adipose cells mainly include WAT and BAT with different biological functions, and WAT has the capacity for browning into BAT or beige characterized by high expression of UCP1 [3,10]. At present, there are no relevant reports on the effects of Ang II on the function and browning of mature adipocytes. Therefore, in this study, we induced 3T3-L1 to differentiate into mature adipocytes before Ang II treatment to observe the effects of Ang II on the function of mature adipocytes. According to our findings, Ang II promoted the browning of mature adipocytes, which was characterized by decreased lipid droplets size, increased lipid droplet and mitochondrial numbers, and increased UCP1 expression (Figure 2C,D). In addition, Ang II significantly affected mitochondrial function in mature 3T3-L1, as characterized by an increase in ATP production, oxygen consumption and mROS generation as well as a markedly decreased MMP (∆Ψm) (Figure 2E–H). Therefore, we suggest that Ang II may promote the browning of mature 3T3-L1 cells and make them produce more energy and heat by regulating mitochondrial numbers and function. This is the first report on the regulatory effect of Ang II on the function of mature adipocytes.

Several nuclear factors, such as PRDM16 and PGC-1α, have been associated with the formation of brown fat cells [9]. In addition, transcriptional repressor CtBP1 is also considered as an important regulator of the brown phenotype [15]. In the current study, the transcription and expression of CtBP1 were dramatically increased after Ang II treatment in mature 3T3-L1 cells (Figure 2A,B). In order to further confirm the role of CtBP1 in Ang II-induced mature 3T3-L1 browning, we knocked out CtBP1 and investigated the browning and mitochondrial function in Ang II-treated mature 3T3-L1 cells. Our results demonstrated that CtBP1 KO significantly inhibited Ang II-induced browning, mitochondrial respiratory function, and mROS production (Figure 2). These findings indicate that the regulatory effects of Ang II on the formation and function of brown fat cells are dependent on CtBP1. It has been reported that the interactions of CtBP1 with PRDM16 or C/EBPα suppress the white genes and induce the brown phenotype [16,24]. Therefore, we speculate that Ang II may inhibit the expression of white genes and regulate the number and function of mitochondria through CtBP1/PRDM16 or CtBP1/C/EBPα interactions, thus promoting and maintaining the brown phenotype.

In conclusion, we found that Ang II affects both the differentiation of 3T3-L1 preadipocytes and the function of mature 3T3-L1 adipocytes through CtBP1. Ang II inhibits 3T3-L1 adipogenicity via CtBP1. In addition, Ang II promotes the browning of mature 3T3-L1 adipocytes through CtBP1, from the function of storing fat to burning fat. Meanwhile, Ang II enhances mitochondrial respiration function, ROS production, and reduces MMP (∆Ψm) via CtBP1 in mature 3T3-L1 adipocytes.

## Data Availability

All data generated during this study are included in this published article and its Supplementary Files.

## Supplementary Materials

The following supporting information can be downloaded at: https://www.mdpi.com/article/10.3390/biomedicines10123131/s1. Supplementary figure legends, Figure S1: A scheme displaying the effects of Ang II and its receptor on 3T3-L1 adipogenic differentiation; Figure S2: A scheme displaying the effects of Ang II on the function of mature adipocytes; Figure S3: The effects of Ang II and its receptor on the expression of adipogenic markers (AP2, PPARγ, C/EBPα, ACC) during 3T3-L1 adipogenic differentiation process. Untreated 3T3-L1 cells were used as control. Histograms show the ratios of target proteins to GAPDH or tubulin. * *p* < 0.05 and ** *p* < 0.01 vs. the control group; # *p* < 0.05 and ## *p* < 0.01 vs. Ang II-treated 3T3-L1 cells (*n* = 3); Figure S4: CtBP1 knockout in 3T3-L1 identified by Western blot analysis. Histogram shows the ratio of CtBP1 to GAPDH. *** *p* < 0.001 vs. the control group (*n* = 3).
