# Supplementary material for: Angiotensin II Inhibits Adipogenic Differentiation and Promotes Mature Adipocyte Browning through the Corepressor CtBP1
Source: Biomedicines. 2022 Dec 4;10(12):3131. doi: 10.3390/biomedicines10123131 (PMC9775054; doi:10.3390/biomedicines10123131)
Supplement: Supplementary file 1 [file biomedicines-10-03131-s001.zip › biomedicines-2027710-supplementary.pdf]

Supplementary Materials

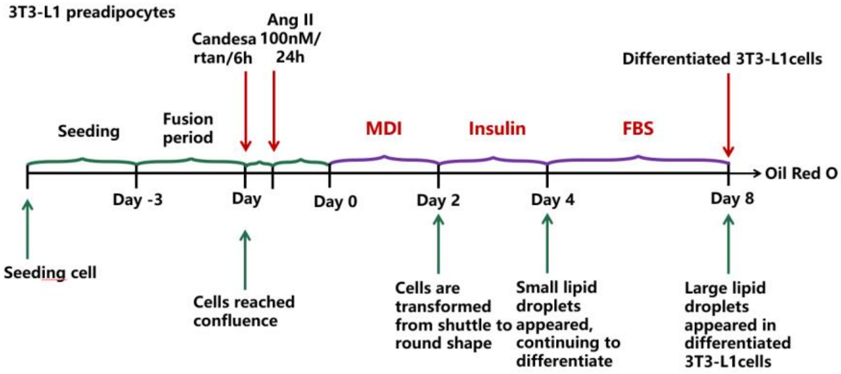

Figure S1. A scheme displaying the effects of Ang II and its receptor on 3T3-L1 adipogenic differentiation.

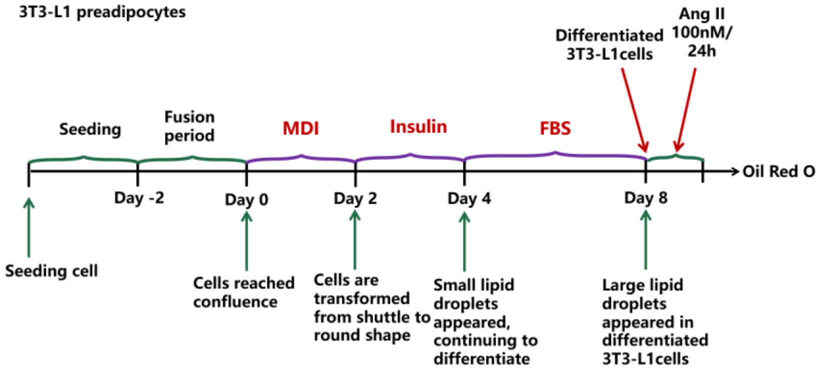

Figure S2. A scheme displaying the effects of Ang II on the function of mature adipocytes.

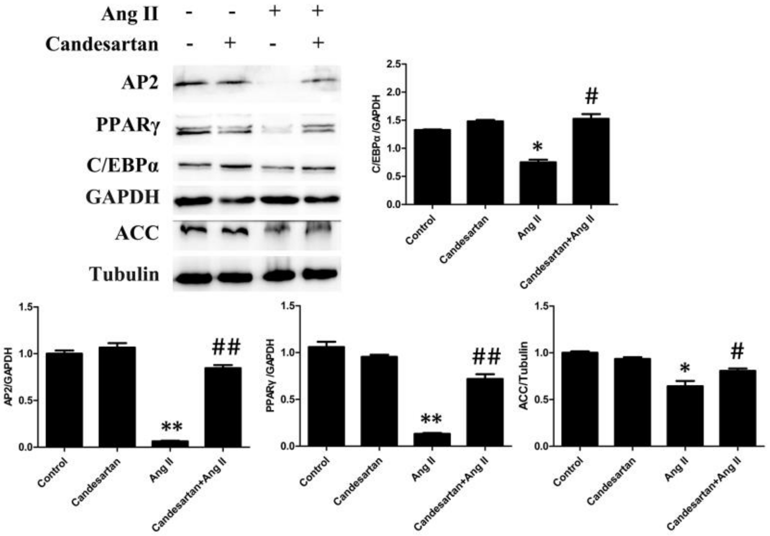

Figure S3. The effects of Ang II and its receptor on the expression of adipogenic markers (AP2, PPARγ, C/EBPα, ACC) during 3T3-L1 adipogenic differentiation process. Untreated 3T3-L1 cells were used as control. Histograms show the ratios of target proteins to GAPDH or tubulin. * $p < 0.05$ and ** $p < 0.01$ vs. the control group; # $p < 0.05$ and ## $p < 0.01$ vs. Ang II-treated 3T3-L1 cells ($n = 3$).

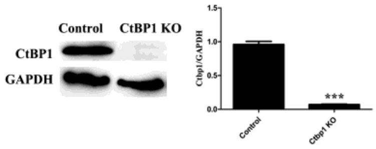

Figure S4. CtBP1 knockout in 3T3-L1 identified by Western blot analysis. Histogram shows the ratio of CtBP1 to GAPDH. *** $p < 0.001$ vs. the control group ($n = 3$).
